# Characterization of the role for cadherin 6 in the regulation of human endometrial receptivity

**DOI:** 10.1186/s12958-020-00624-w

**Published:** 2020-06-29

**Authors:** Wei Zhou, Leilani Santos, Evdokia Dimitriadis

**Affiliations:** 1 Department of Obstetrics and Gynaecology, University of Melbourne, Parkville, Victoria 3010 Australia; 2 Gynaecology Research Centre, Royal Women’s Hospital, Parkville, Victoria 3052 Australia

**Keywords:** Endometrial receptivity, Adhesive molecules, CDH6, Embryo implantation, Endometrial epithelial cell, Trophoblast cell

## Abstract

**Background:**

The endometrial luminal epithelium is the first point of attachment of embryos during implantation. Failure of embryos to firmly adhere results in implantation failure and infertility. A receptive endometrial luminal epithelium is achieved through the expression of adhesion molecules in the mid-secretory phase and is a requirement for implantation. Cadherin 6 (CDH6) is an adhesion molecule localizing to the endometrial luminal epithelial cell surface in the mid-secretory/receptive phase and knockdown of *CDH6* in the Ishikawa cells (receptive endometrial epithelial cell line) compromises cell integrity. However, there are no studies investigating the role of CDH6 on receptivity and infertility. This study aimed to investigate whether CDH6 is dysregulated in the endometrium of women with infertility during the receptive window and the effect of CDH6 on endometrial adhesion and receptivity.

**Methods:**

The expression and the localization of CDH6 in the human endometrium were determined by immunohistochemistry. Ishikawa cells were used to investigate the functional consequences of *CDH6* knockdown on endometrial adhesive capacity to HTR8/SVneo (trophoblast cell line) spheroids in vitro. *CDH6* knockdown was assessed by qPCR and immunoblotting. After *CDH6* knockdown, the expression of type II cadherin family members and CDH6 functional partners were assessed by qPCR. Two-tailed unpaired student’s t-test or one-way ANOVA as appropriate were used for statistical analysis with a significance threshold of *P* < 0.05.

**Results:**

A significant reduction of CDH6 immunolocalization was recorded in the luminal and glandular epithelium of endometrium from women with infertility (*P* < 0.05) compared to fertile group respective cellular compartments in the mid-secretory phase. Functional analysis using Ishikawa cells demonstrated that knockdown of *CDH6* (treated with 50 nM *CDH6* siRNA) significantly reduced epithelial adhesive capacity (*P* < 0.05) to HTR8/SVneo spheroids compared to control and other type II cadherin family members likely failed to compensate for the loss of CDH6. The expression levels of CDH6 functional partners, catenin family members were not changed after *CDH6* knockdown in Ishikawa cells.

**Conclusion:**

Together, our data revealed that CDH6 was dysregulated in the endometrium from women with infertility and altered Ishikawa cell adhesive capacity. Our study supports a role for CDH6 in regulating endometrial adhesion and implantation.

## Background

Infertility affects a staggering 1 in 10 couples of reproductive age worldwide [1]. A failure of embryo implantation is a major cause of infertility. Human embryo implantation starts with the initial contact and adhesion between a blastocyst and the endometrial luminal epithelium [2, 3]. The inadequate adhesive capacity of the endometrial luminal epithelium leads to inadequate embryo attachment and implantation failure. Defective endometrial adhesion is one of the leading causes of implantation failure and infertility [4, 5] while the mechanisms contributing to this remain poorly defined.

The endometrial luminal epithelium is only receptive to an implanting blastocyst within a narrow window of 2–4 days in the mid-secretory phase [6, 7]. Within this time frame, the luminal epithelium undergoes a complex series of changes including the altered expression of adhesive molecules such as cadherins to ensure embryos attach and firmly adhere to initiate implantation [8]. Cadherins comprise a large family of cell adhesion molecules consisting of more than eighty family members [9] and are key regulators of cell adhesion, sorting and invasion [10]. Structurally, cadherins contain repeated extracellular domains that interact generally in a homophilic manner to their own family members to mediate cell-cell interactions, whereas their cytoplasmic regions are associated with a wide range of proteins including cytoskeletal regulators, enzymes and transcriptional factors which endow them with diverse downstream functional capabilities [10].

A previous high-density microarray screen on human endometrium reveals an increase in the expression of cadherins and associated functional partners in mid-secretory phase, compared to the early-secretory phase [11]. The localization of the three classical cadherin members, E-cadherin, N-cadherin and P-cadherin have been well characterized. The expression of these three cadherins are mainly restricted to the epithelial cell in the secretory phase, although their localisation specifically in the endometrial luminal epithelium is not reported [12, 13]. Further studies have revealed a potential dual function in facilitating embryo implantation [14, 15]. As adhesive molecules, the apical surface expression of cadherins can directly contribute to mechanical cell-cell adhesion. An in vitro functional study in a non-receptive endometrial epithelial cell line AN3-CA demonstrated that forced overexpression of E-cadherin in these cells significantly increased their receptivity to trophoblast-like spheroids formed by the BeWo choriocarcinoma cells [16]. By contrast, downregulation of E-cadherin in the lateral surface of the uterine epithelium in both human (in vitro) and mouse (in vivo) models may serve as a key mechanism to facilitate the loss of apical-basal polarity in the epithelial layer [17, 18]. Such change is likely to avoid mutual repulsion between the embryo and the polarized endometrial luminal epithelial surface to facilitate embryo attachment and invasion [19].

Limited studies have investigated the localization and function of type II cadherin family members in the human endometrium. CDH11 is a predominant cadherin subtype in endometrial stromal cells in the secretory phase [20]. CDH5 is expressed in the late proliferative phase in endometrial explants [21] and in endometrial mesenchymal stromal cells [22]. An in vitro study demonstrated that expression of CDH5 in the mouse trophectoderm facilitates embryo implantation [23]. A previous characterization study revealed that CDH6 expression is decreased in the glandular epithelium and stromal cells in the receptive phase with no information available on the localization and expression in the luminal epithelium [24]. Another recent study confirmed that CDH6 immunolocalizes to the apical and lateral cell borders in the endometrial luminal epithelium in the mid-secretory phase [25]. Further assessment using a receptive endometrial epithelial cell line, Ishikawa cells, indicates that knockdown of *CDH6* in Ishikawa cells at high siRNA concentrations (50 and 100 nM) impact the integrity of Ishikawa cell monolayers compared to low siRNA concentrations (10 and 20 nM) or control siRNA [25].

To the best of our knowledge, there is no research exploring whether CDH6 plays a role in regulating endometrial epithelial cell adhesive capacity and receptivity and whether it is dysregulated in the endometrium of women with infertility during the receptive window. We examined the clinical relevance of CDH6 on receptivity by determining CDH6 immunostaining levels in mid-secretory phase endometrium from fertile and infertile patients. We used the Ishikawa cells as an in vitro model of endometrial epithelial cells to determine whether siRNA knockdown of *CDH6* compromised their adhesive capacity to HTR8/SVneo trophoblast spheroids. It has been previously identified that in neurons, other cadherins can compensate for the loss of CDH6 to maintain the correct positioning of neurons in the mouse model [26]. We thus also investigated the effect of *CDH6* knockdown on the expression of other type II cadherin family members and CDH6 functional partners in Ishikawa cells.

## Methods

### Antibodies and cell lines

Rabbit polyclonal antibody against CDH6 (HPA007456) was purchased from Sigma (St. Louis, MO, USA). Horseradish Peroxidase (HRP) conjugated rabbit monoclonal antibody against Glyceraldehyde 3-phosphate dehydrogenase (GAPDH, #3683) was from Cell Signaling Technology (Danvers, MA, USA). The Ishikawa cell line was provided by Dr. M. Nishida (Tsukuba University, Tochigi, Japan). The Ishikawa cell line is a well-established in vitro model of primary receptive endometrial epithelial cells derived from human endometrial adenocarcinoma cells that exhibits similar characteristics to endometrial luminal and glandular epithelial cells [27]. Besides the expression of hormone receptors being similar to normal primary endometrial cells [28], Ishikawa cells also possess apical adhesiveness and are appropriate to study endometrial receptivity and embryo attachment [27]. The HTR8/SVneo trophoblast cell line (CRL-3271) was purchased from the ATCC and cultured as in the manufacturer’s instructions.

### Endometrial tissue collection

Written informed consent was obtained from each patient before surgery, with protocols approved by the Human Research Ethics Committee at Monash Health (ID: #03066B) and the Royal Women’s Hospital (SSA1813). All women (26–42 years of age) consented had regular menstrual cycles (28–32 day), were not using intrauterine contraceptives and had not used hormones for at least 3 months before surgery. Fertile women had proven parity (≥1 parous pregnancy) and were having surgery for mirena insertion, benign ovarian cyst assessment or polypectomy. Infertile women had primary unexplained infertility defined as being unable to conceive after one-year and had no apparent endometrial dysfunction diagnosed using routine fertility investigations (hysteroscopic and laparoscopic diagnosis, including endometriosis, endometritis or other endometrial related disorders). Partners of the infertile group had normal sperm analysis including sperm counts, motility and morphology. The endometrial tissue samples were collected by curettage and were from the functionalis layer. The collected endometrium were examined by gynecological pathologists based at the Royal Women’s Hospital to confirm the cycle stage and absence of endometrial dysfunction.

### *CDH6* siRNA transfection

We transfected the Ishikawa cells with three different concentrations of *CDH6* siRNA based on a previous study [25]. Ishikawa cells at 70–80% confluency were transfected with Lipofectamine RNAiMAX and Opti-MEM medium (Thermo, Waltham, MA, USA) containing *CDH6* siRNA (10, 20 or 50 nM) or scrambled control (50 nM) (Dharmacon, Lafayette, CO, USA) according to the manufacturer’s instructions. Before transfection, cells were washed three times with phosphate-buffered saline (PBS) to remove antibiotics. After 24 h the transfection medium was replaced with fresh culture medium and Ishikawa cells were cultured for 48 h before being subjected to spheroid adhesion assay or other downstream analyses.

### Immunohistochemistry and immunocytochemistry

Human fertile and infertile endometrial tissues (mid-secretory phase defined as days 20–24 of an idealized 28-day menstrual cycle) [29] were fixed in 10% formalin, embedded in paraffin and sectioned at 4 μm thickness. Slides were dewaxed, rehydrated and subjected to antigen retrieval under optimized condition (microwaving in 10 mM sodium citrate for 5 min). Endogenous peroxidase was blocked using 3% hydrogen peroxide in methanol for 15 min. Slides were then incubated with non-immune blocking containing 10% goat serum and 2% human serum in Tris-buffered saline (TBS) for 45 min at room temperature (RT). CDH6 antibody was added at the concentration of 0.25 μg/mL overnight at 4 °C. Equivalent isotype control (Dako, X0903) was included in which sections were incubated with a normal rabbit immunoglobulin fraction at the same concertation (0.25 μg/mL). Slides were washed with TBS-Tween 0.6% (v/v) and positive signaling was revealed via the avidin-biotin- diaminobenzidine system. Sections were counterstained with haematoxylin to indicate cell nuclei (blue). Slides were then mounted with DPX and imaged using an Olympus light microscope. Staining intensity scores were determined by two individual scorers blinded to the patient characteristic, as previously described [30]. Briefly, a score of 0 denoted no CDH6 staining and 3 was maximal staining. Each score was based on overall staining intensity of the whole endometrial section. Scores were analyzed and plotted using Graphpad Prism 7. For immunocytochemistry staining of cell cultures, the cells were settled onto Ibidi μ-slide culture chambers. Cells were then fixed in 4% paraformaldehyde for 15 min and permeabilized by incubation in 0.1% Triton X-100 for 10 min. After PBS wash, cells were immunolabeled as described for endometrial sections.

### Spheroid adhesion assay

HTR8/SVneo cells were plated in a U shape and ultra-low attachment 96 well plate (Corning, NY, USA) and cultured for 48 h to form spheroids (2000 cells/spheroid). Spheroids were then harvested and transferred to transfected Ishikawa cell monolayer (20 spheroids/well of 96 well plate) to initiate spheroid adhesion assay. Briefly, original spheroid number in each well was determined using a light microscope before been subjected to 4 h co-culture with Ishikawa monolayer. Following incubation, each well was gently washed once by adding 150 μL PBS to remove non-adherent spheroids. The remaining spheroids (adhered spheroids) were counted and attachment was expressed as a percentage of the original spheroid number as previously reported [31].

### RNA isolation and RT-q-PCR

Ishikawa cells were lysed with TRI Reagent (Sigma). RNA was isolated according to the manufacturer’s protocol and treated with RNase-free DNase set (Qiagen, Germantown, MD, USA) to remove genomic DNA contamination. RNA concentration was determined by Nanodrop spectrophotometers (Thermo). For reverse transcription, 300 ng total RNA was converted to cDNA using SuperScript™ III First-Strand Synthesis System (18080–051, Thermo). qPCR was performed on the Applied Biosystems ViiA7 system using SYBR Green Master Mix (4,367,659, Thermo) as follows: 95 °C for 10 min and 40 cycles of 95 °C for 15 s followed by 60 °C for 1 min. Primers used were summarized in Additional file 2. Gene expression was normalized to *18S*. Relative expression levels were calculated using the comparative cycle threshold method (ΔΔCt).

### SDS-PAGE and immunoblotting

The organic phase from TRI Reagent based RNA isolation was collected for protein extraction with the detailed protocol optimized by a previous study [32]. Proteins were then resolved by SDS-PAGE (150 V, 1 h) and transferred to PVDF membranes (100 V, 1 h). Membranes were then blocked with 5% skim milk and incubated with the CDH6 antibody (1:1000) overnight at 4 °C. Membranes were washed with TBS-Tween 0.1% (v/v) and incubated with HRP conjugated secondary antibody against rabbit and HRP-GAPDH (1:3000, as a loading control). After three additional washes, labeled proteins (CDH6 and GAPDH) were detected by chemiluminescence (Thermo).

### Statistics

Statistical analysis was performed using PRISM 8.0 and two-tailed unpaired student’s t-test or one-way ANOVA as appropriate with a significance threshold of *P* < 0.05. Data were presented as the mean ± SEM.

## Results

### CDH6 expression is reduced in the mid-secretory phase endometrium from women with infertility, compared to fertile group

**Fig. 1:**
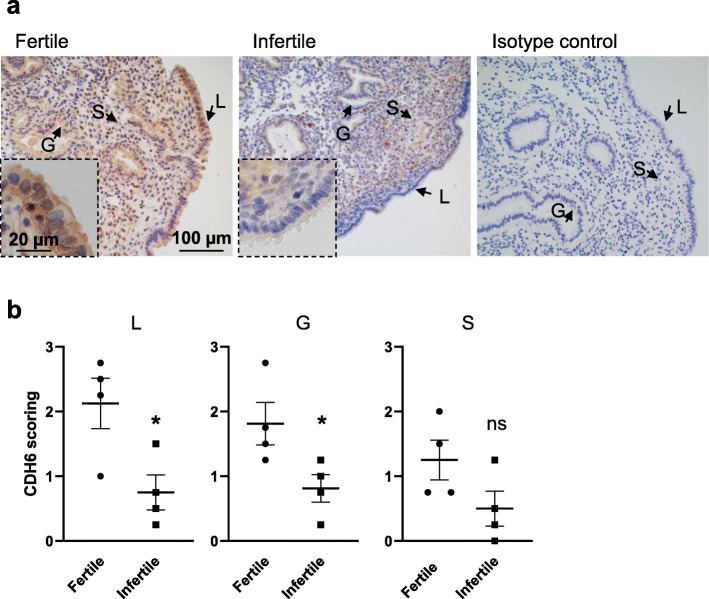
Comparison of CDH6 immunolocalization in fertile and infertile mid-secretory phase endometrium. **a** CDH6 immunolocalized to the luminal epithelium (L), glandular epithelium (G) and stromal cells (S). Higher magnification images are outlined. The specificity of CDH6 labeling was confirmed through the inclusion of an isotype control in which the non-immune antibody of the same isotype was substituted for the CDH6 antibody at the same concertation. Sections were counterstained with hematoxylin to indicate the cell nuclei (blue). **b** Staining intensity of CDH6 was semi-quantitated by scoring staining in tissues blinded to fertility status. Data were presented as mean ± SEM. (*n* = 4). *P < 0.05, ns: no significant difference

To investigate the functional significance of CDH6 in the human endometrium, we first sought to determine the clinical relevance of this target in infertility by assessing its immunolocalization in mid-secretory phase endometrium from women with normal fertility (fertile) and primary infertility (infertile). In our immunostaining analysis of fertile endometrium, in addition to membrane staining, CDH6 was also localized to the cytoplasm of the fertile endometrium (Fig. 1a). By contrast, the infertile endometrium showed a reduced level of CDH6 staining in the luminal and glandular epithelium compared to the fertile group. Semi-quantification of the CDH6 staining intensity confirmed the reduction to be significant (*P* < 0.05) in both luminal and glandular epithelium. No significant difference was found in the stromal cells between fertile and infertile groups (Fig. 1b). The specificity of CDH6 labeling was confirmed by the inclusion of an isotype control and as expected, no staining was revealed (Fig. 1a).

### Knockdown of *CDH6* in the Ishikawa cells impaired HTR8/SVneo spheroid adhesion

**Fig. 2:**
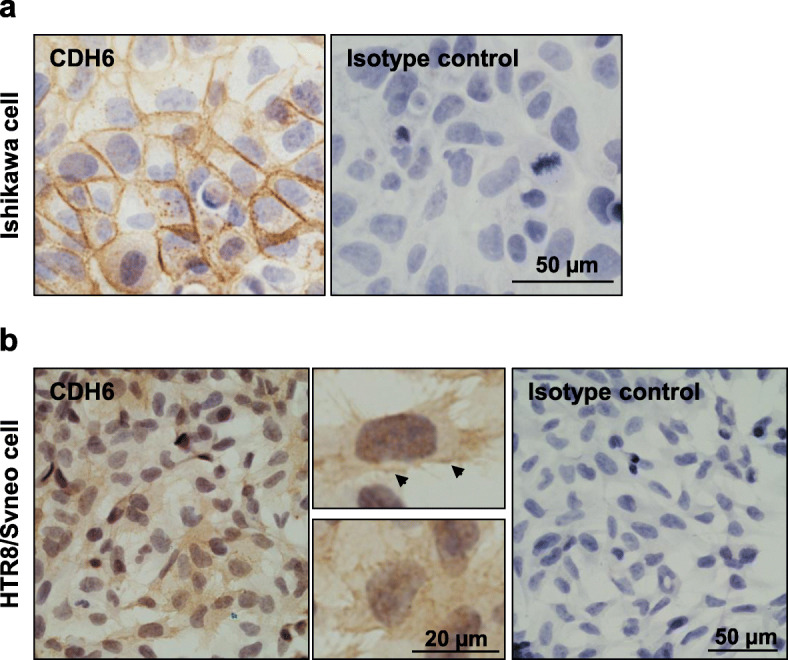
Immunocytochemistry detection of CDH6 in the Ishikawa cells and HTR8/SVneo cells. **a** In Ishikawa cells, CDH6 localization was detected to the plasma membrane with moderate staining appearing in the cytoplasm. **b** A similar localization was observed in the HTR8/SVneo cells (arrows) with extra staining also found in the cytoplasm. Higher magnification images of CDH6 localization in HTR8/SVneo cells are depicted on the right of panels. The specificity of CDH6 labeling was confirmed through the inclusion of an isotype control as described in Fig. 1. Sections were counterstained with hematoxylin to indicate the cell nuclei (blue)

We next used Ishikawa cells in combination with HTR8/SVneo spheroids to determine the functional consequences of *CDH6* knockdown on endometrial epithelial adhesive capacity. Prior to use, these cells were assessed for their expression of CDH6 by immunocytochemistry. Consistent with the localization of CDH6 in the fertile endometrium (Fig. 1a), CDH6 localized to the membrane of Ishikawa cells with moderate staining appearing in the cytoplasm (Fig. 2 a). Based on the similar expression patterns compared to luminal epithelium in the fertile endometrium, the Ishikawa cells were deemed a suitable model to explore CDH6 function. We also demonstrated for the first time that in HTR8/SVneo cells, CDH6 localized to the cell cytoplasm and membrane (Fig. 2b).

**Fig. 3:**
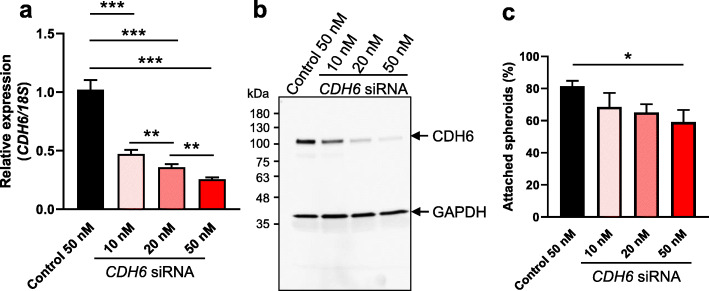
Examination of the effect of *CDH6* knockdown on Ishikawa cell adhesive capacity. Ishikawa cells were transfected with either scrambled control (50 nM) or *CDH6* siRNA (10, 20 or 50 nM) before HTR8/SVneo spheroid adhesion assay. **a***CDH6* knockdown was determined by qPCR. Expression levels were normalized to *18S* (*n* = 7). **b** Immunoblotting was also used to determine the CDH6 expression. Blots were co-probed with an anti-GAPDH antibody to confirm equivalent protein loading of each sample. **c** A siRNA concentration-dependent reduction of the adhesion was observed in Ishikawa cells with the highest concentration of *CDH6* siRNA (50 nM) significantly compromised the spheroid adhesion compared to scrambled control. Data were presented as mean ± SEM. *P < 0.05, **P < 0.01, ***P < 0.001

We transfected the Ishikawa cells with three different concentrations of *CDH6* siRNA. Our qPCR data confirmed that *CDH6* siRNA treatment at all concentrations significantly reduced *CDH6* expression compared to scrambled control (Fig. 3a, *P* < 0.001). The extent of CDH6 reduction was siRNA concentration dependent, with the lowest expression of *CDH6* being recorded in the Ishikawa cells that had the highest concentration of *CDH6* siRNA treatment (Fig. 3a). Immunoblotting was also used to assess the CDH6 protein levels after *CDH6* siRNA treatments and similar to the effect on *CDH6* mRNA, we confirmed the knockdown of CDH6 protein after siRNA treatment at the different concentrations (Fig. 3b). Following knockdown of *CDH6* in the Ishikawa cells, HTR8/SVneo spheroids were added and their adhesive capability on the Ishikawa monolayer was determined. As shown in Fig. 3c, Ishikawa cells transfected with *CDH6* siRNA at 50 nM had significantly reduced spheroid adhesion compared to scrambled control (*P* < 0.05). No significant difference in spheroid adhesion was observed with *CDH6* siRNA treatment at 10 and 20 nM compared to scrambled control. Notably, no discernible effect on Ishikawa cell monolayer integrity / morphology was recorded with *CDH6* siRNA treatment at 50 nM which is in contrast to a previous report (Additional file 1).

### The effect of *CDH6* knockdown on the expression of *CDH* family members and functional partners

**Fig. 4:**
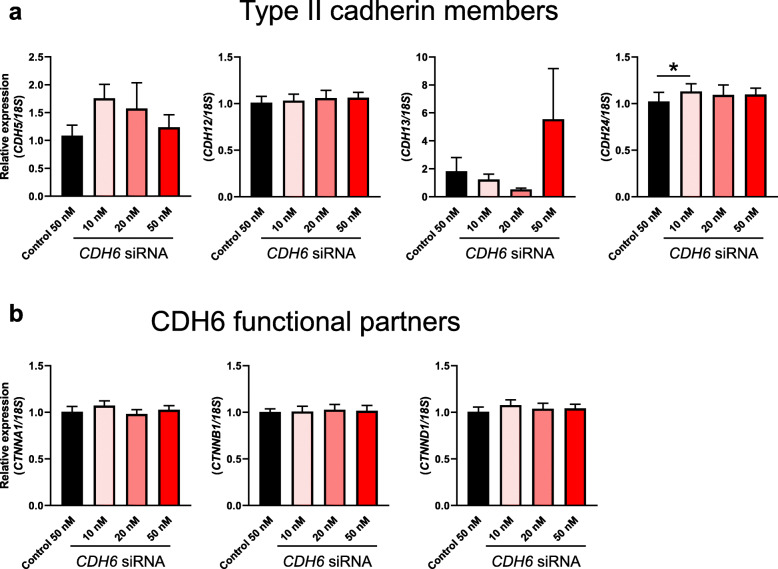
Examination of the effect of *CDH6* knockdown on the expression of other targets in Ishikawa cells: (**a**) other Type II cadherin family members and (**b**) CDH6 functional partners. Expression levels were normalized to *18S* (*n* = 6). Only *CDH24* expression was significantly increased after *CDH6* knockdown (at 10 nM siRNA treatment) compared to scrambled control. Data were presented as mean ± SEM. *P < 0.05

Since previous studies have shown that cadherin family members share moderate levels of functional redundancy [33, 34], it is possible that other type II cadherin members may compensate for the loss of CDH6 in the Ishikawa cells as no significant effect on HTR8/SVneo spheroid adhesion was recorded at 10 and 20 nM *CDH6* siRNA treatments compared to scrambled control (Fig. 3c). The expression of type II cadherin members in Ishikawa cells following *CDH6* knockdown was explored by qPCR. This analysis indicated that in scrambled control treated Ishikawa cells, *CDH12* and *CDH24* were expressed at relatively high levels (average raw Ct value 26) compared to *CDH5* and *CDH13* (average raw Ct value 32–33) (Additional file 3). *CDH11* expression was undetectable in Ishikawa cells (data not shown). Among all the type II cadherin family members examined, *CDH6* knockdown in Ishikawa cells had no significant effect on the expression levels of *CDH5*, *CDH12* and *CDH13* compared to scrambled control while *CDH24* expression was only significantly increased with *CDH6* siRNA treatment at 10 nM (Fig. 4a, *P* < 0.05). *CDH6* reduction in the Ishikawa cells did not affect the expression levels of the CDH6 functional partners, Catenin family members including Catenin alpha 1 (*CTNNA1*), Catenin beta 1 (*CTNNB1*) and Catenin delta 1 (*CTNND1*) (Fig. 4b).

## Discussion

Adhesive proteins in the endometrial luminal epithelium fulfill essential roles in facilitating embryo attachment. In this study, we demonstrated for the first time that CDH6 reduction was associated with infertile endometrium during the receptive window. We used Ishikawa cells as an in vitro model to explore the functional consequences of *CDH6* knockdown on their adhesive capacity to HTR8/SVneo spheroids. We demonstrated that knockdown of *CDH6* significantly reduced the spheroid adhesion compared to scrambled control under 50 nM condition and did not affect the expression of other type II cadherin family members or CDH6 functional partners, indicating CDH6 has a non-redundant role in regulating endometrial receptivity.

CDH6 belongs to type II classical cadherin and it regulates cell adhesion via a homophilic binding to its own family members. In order to regulate embryo adhesion, cadherin family members are required to be expressed in both endometrial epithelial cells and blastocyst trophectoderm. qPCR analysis from our study revealed that all type II cadherin family members were expressed in the Ishikawa cells. Gene expression analysis of trophectoderm isolated from preimplantation human embryos demonstrates all type II cadherin family members are expressed in the trophectoderm [35]. This indicates that all type II classical cadherins are able to directly regulate embryo adhesion onto the endometrial luminal epithelium. Our data showed that although lower concentrations of *CDH6* siRNA treatment (10 and 20 nM) in the Ishikawa cells did not compromise spheroid adhesion, 50 nM *CDH6* siRNA treatment significantly reduced their adhesive capacity to HTR8/SVneo spheroids suggesting that either the level of CDH6 was critical for adhesion or the existence of other type II classical cadherins were not able to compensate for the severe loss of CDH6. It is not unusual that cadherin family members play non-redundant roles in cell adhesion and invasion. A previous study in mouse embryos reveals a unique role of E-cadherin in regulating the formation of polarized functional trophectoderm by demonstrating that the replacement of E-cadherin by N-cadherin is not sufficient to form an intact trophectoderm [36]. After embryo attachment, the trophectoderm differentiates into trophoblast and starts to invade into the uterus. It has been revealed that in the invasive front of extravillous cytotrophoblasts, CDH6 is the predominant cadherin subtype and its expression promotes the cell invasive capacity [20, 37].

This leads to an intriguing notion of cadherin expression that has been recorded during epithelial to mesenchymal transition in embryonic development, namely the cadherin switch [38]. During this process, the expression of the epithelial cell marker E-cadherin is reduced whereas the expression of another cadherin family member N-cadherin is increased. Such an expression switch coincides with a morphological and phenotypic transition of the pre-migrating cells [39]. A similar change in cadherin expression has also been recorded in tumor cells. Loss of E-cadherin and increased expression of N-cadherin and CDH11 in the human prostate cancer cells is able to change the invasive capacity and metastasis of the cells [40]. In the mid-secretory phase human endometrium, in contrast to the localization of CDH6 in the apical and lateral surface of endometrial luminal epithelium, E-cadherin protein expression is reduced and shows minimal levels [41]. Such an expression reduction has been proven essential for embryo implantation and invasion in mice [17]. It is likely that selective changes in expression of E-cadherin and CDH6, along with other notable cadherin family members that have been reported in the human endometrium [12, 13, 20] contribute to a functional transition that is required for successful embryo implantation.

A particular curiosity of this hypothesis is how the differential expression of the same cadherin family members lead to a functional transition in endometrial epithelial cells. One possible explanation is that different family members may have different isoforms. The isoform differences enable even the same cadherin family member to have different functional capacities. It has recently been demonstrated that CDH6 has two isoforms that are inherently different: CDH6-long isoform and CDH6-short isoform [37]. The difference between these two isoforms is that the CDH6-short isoform does not have an intracellular domain to interact with its functional partners [37] and it may lose the ability to activate downstream signaling pathways. It is likely that this isoform only functions as an adhesive molecule during cell-cell interactions. The antibody we used in this study binds to the extracellular domain thus recognizes both isoforms. We remain uncertain if these two isoforms co-exist in the endometrial luminal epithelium to regulate different functional aspects of the implantation process. Different isoforms of cadherin family members may fulfill specific functional requirements within a specific cell type or development stage.

Previous studies have also proposed a cellular ‘redistribution’ theory that may contribute to the functional complexity of the cadherins. In the human endometrium, the lateral surface expression of CDH6 is essential to form the adherens junctions to maintain the integrity of the endometrial luminal epithelium before implantation. Once the embryo contacts the endometrial luminal epithelium, the lateral adherens junction proteins such as CDH6 may redistribute to the apical surface to facilitate the attachment of the embryo and such redistribution may break down the adherens junctions to allow or facilitate embryo invasion [25, 42]. The redistribution of CDH6 also releases the cadherin functional partners such as catenin proteins that are required to form adherens junctions. Another role of catenin proteins is to participate in Wnt/β-catenin (CTNNB1) signaling that is essential for implantation in vivo in mice [43]. Wnt/β-catenin (CTNNB1) signaling activation similarly requires the local stimulus of the embryo [43] which supports the ‘redistribution’ theory. Our immunohistochemistry data revealed that CDH6 was downregulated in the apical and lateral surfaces of the endometrial luminal epithelium in the infertile endometrium during the mid-secretory phase suggesting CDH6 affects both adhesion and integrity of the endometrial luminal epithelium.

Several mechanisms can reduce CDH6 expression in the infertile endometrium. microRNA downregulates gene expression [44] and our previous studies reveal that specific microRNAs are increased in the infertile human endometrium and affect endometrial adhesive capacity and receptivity by downregulating essential gene targets [30, 31]. *CDH6* is a direct gene target of miR-223-3p and forced overexpression of miR-223-3p in cultured osteosarcoma cells reduces *CDH6* expression and inhibits cell invasion and migration [45]. miR-223-3p also suppresses the expression of leukemia inhibitory factor (*LIF*) during the implantation window in mouse uterus [46], a target that is essential for embryo implantation in both humans and mice [47, 48]. A previous study confirms the upregulation of miR-223-3p in human endometrium with a compromised receptivity phenotype and its interaction with *LIF* [49]. In support, in our recent study we reveal that miR-223-3p is detected at low levels in the primary fertile human endometrial epithelial cells [50]. However, whether *CDH6* is directly regulated by miR-223-3p in the infertile human endometrium is unknown. Similarly, *CDH6* may be downregulated in infertile endometrium epigenetically by DNA methylation. An in vitro study on human endometrial epithelial cell line AN3-CA has confirmed that E-cadherin gene expression is negatively controlled by DNA methyltransferase enzymes. Inhibition of these enzymes increases the expression of E-cadherin and switch the non-receptive endometrial epithelial cells to become receptive to trophoblast-like spheroids formed by the BeWo choriocarcinoma cells [16]. In support, an in vivo mouse model has revealed that increased DNA methylation on the promoter of homeobox A10 (*Hoxa10*) reduces the expression of *Hoxa10* and directly affects endometrial receptivity [51]. There are no studies that have investigated whether the downregulation of *CDH6* is related to increased DNA methylation in any biological system. Whether this is the case in the infertile endometrium warrants further investigation.

Ishikawa cells transfected with 50 nM of *CDH6* siRNA did not compromise cell integrity which is in contrast to a previous report [25]. This may have been due to methodological differences between the two studies. One notable difference in our study is that we transfected the Ishikawa cells at 70–80% confluency while in the previous report the cells were transfected at 40–50% confluency. After transfection we cultured the Ishikawa cells for 48 h while in the previous study, the Ishikawa cells were cultured for up to 120 h which may have severely impacted the cell integrity.

## Conclusions

In conclusion, our study has provided evidence that CDH6 was abnormally reduced in infertile endometrium during the receptive window. Functional analysis demonstrated that CDH6 may play a non-redundant role in the regulation of endometrial receptivity. Further studies are encouraged to use primary endometrial epithelial cells to confirm these results. Overall our study suggests that CDH6 may be useful as a biomarker or treatment target for dysregulated receptivity in women with infertility.

## Supplementary information

**Additional file 1. ***CDH6* siRNA treatment at 50 nM has no discernable effect on Ishikawa cell integrity. After adhesion assay, attached spheroids on the Ishikawa cell monolayer were indicated with arrows. A higher magnification image of the Ishikawa cells was depicted on the right panel with its original location indicated by outlines on the left.

**Additional file 2.** Primers used throughout this study.

**Additional file 3. **Detection of Type II cadherin family members and CDH6 functional partners in the Ishikawa cells. Expression levels were assessed by raw Ct value (*n* = 6).

## Data Availability

All data generated through this study are included in this article.

## Abbreviations

CDH6: Cadherin 6
HRP: Horseradish peroxidase
GAPDH: Glyceraldehyde 3-phosphate dehydrogenase
TBS: Tris-buffered saline
PBS: Phosphate-buffered saline
RT: Room temperature
LIF: Leukemia inhibitory factor
Hoxa10: Homeobox A10
