# Supplementary figures and images for: Characterization of the role for cadherin 6 in the regulation of human endometrial receptivity
Source: Reprod Biol Endocrinol. 2020 Jun 29;18:66. doi: 10.1186/s12958-020-00624-w (PMC7322878; doi:10.1186/s12958-020-00624-w)

## Slide 1
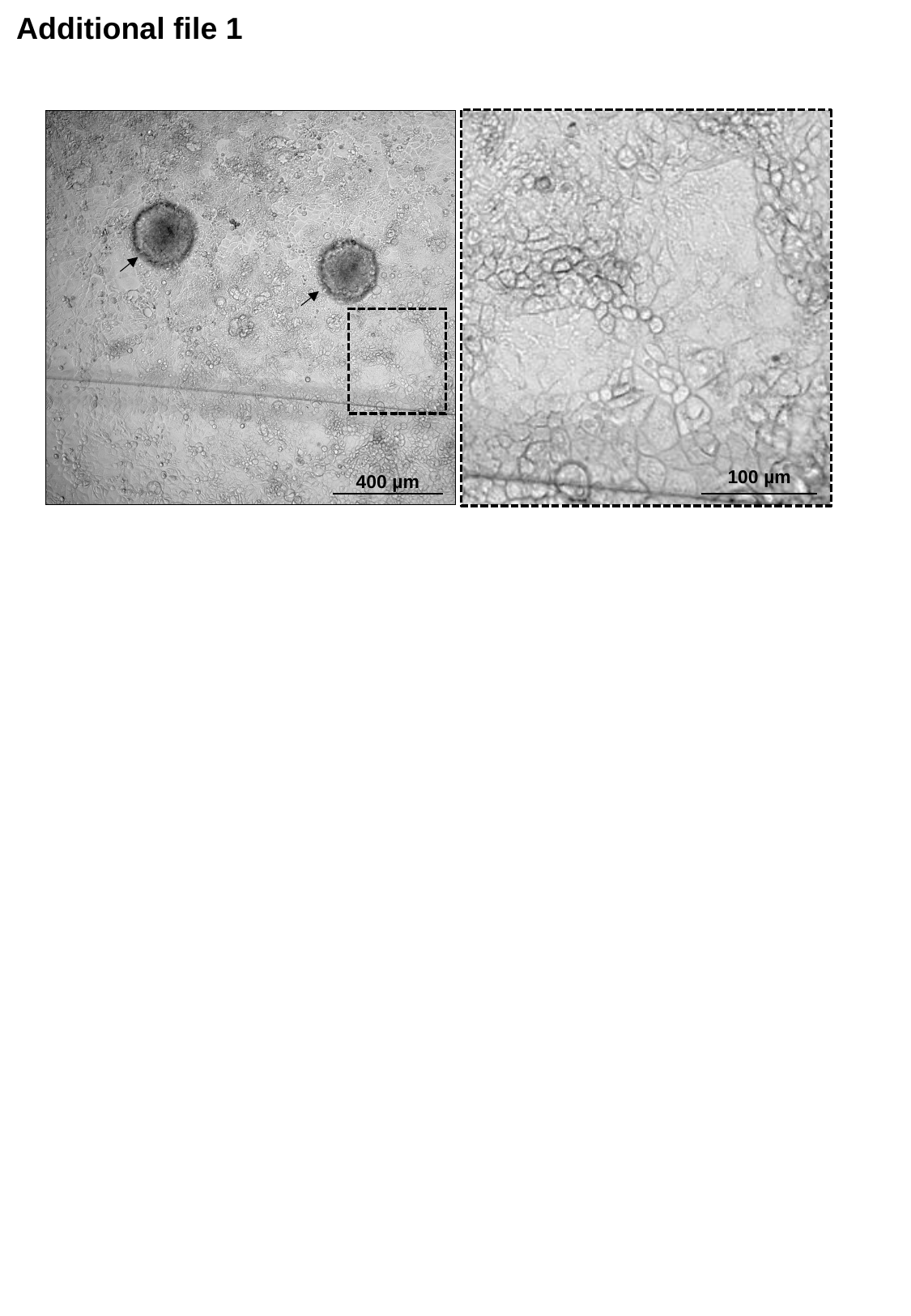

Additional file 1
100 µm
400 µm

Supplement: Supplementary file 1 — Additional file 1. CDH6 siRNA treatment at 50 nM has no discernable effect on Ishikawa cell integrity. After adhesion assay, attached spheroids on the Ishikawa cell monolayer were indicated with arrows. A higher magnification image of the Ishikawa cells was depicted on the right panel with its original location indicated by outlines on the left. [file 12958_2020_624_MOESM1_ESM.pptx]
