# Supplementary material for: Characterization of the role for cadherin 6 in the regulation of human endometrial receptivity
Source: Reprod Biol Endocrinol. 2020 Jun 29;18:66. doi: 10.1186/s12958-020-00624-w (PMC7322878; doi:10.1186/s12958-020-00624-w)
Supplement: Supplementary file 2 — Additional file 2. Primers used throughout this study. [file 12958_2020_624_MOESM2_ESM.docx]

| **Additional file 2. Primers used throughout this study.** | | |
| --- | --- | --- |
| **Gene** | **Forward (5'-3')** | **Reverse (5'-3')** |
| *CDH6* | ACCCAGTTCAAAGCAGCACT | GCAAACAGCACCACTGTCAC |
| *CDH5* | TTGGAACCAGATGCACATTGAT | TCTTGCGACTCACGCTTGAC |
| *CDH12* | TTTGATGGAGGTCTCCTAACACC | ACGTTTAACACGTTGGAAATGTG |
| *CDH13* | AGTGTTCCATATCAATCAGCCAG | CCTTACAGTCACTGAAGGTCAAG |
| *CDH24* | CAGGCACCGTATTTGTGATTGA | GTAGCACATATTGCGCCTTTTC |
| *CTNNA1* | GGGGATAAAATTGCGAAGGAGA | GTTGCCTCGCTTCACAGAAGA |
| *CTNNB1* | CATCTACACAGTTTGATGCTGCT | GCAGTTTTGTCAGTTCAGGGA |
| *CTNND1* | GTGACAACACGGACAGTACAG | TTCTTGCGGAAATCACGACCC |
| *18S* | GATCCATTGGAGGGCAAGTCT | CCAAGATCCACCTACGAGCTT |
