# Supplementary material for: Characterization of the role for cadherin 6 in the regulation of human endometrial receptivity
Source: Reprod Biol Endocrinol. 2020 Jun 29;18:66. doi: 10.1186/s12958-020-00624-w (PMC7322878; doi:10.1186/s12958-020-00624-w)
Supplement: Supplementary file 3 — Additional file 3. Detection of Type II cadherin family members and CDH6 functional partners in the Ishikawa cells. Expression levels were assessed by raw Ct value (n = 6). [file 12958_2020_624_MOESM3_ESM.docx]

**Additional file 3. Detection of Type II cadherin family members and CDH6 functional partners in the Ishikawa cells.**

| **Gene** | **Average raw Ct value** |
| --- | --- |
| *CDH6* | 21.009 |
| **Type II classical cadherin** | |
| *CDH5* | 32.418 |
| *CDH12* | 26.05145264 |
| *CDH13* | 33.204 |
| *CDH24* | 26.99939283 |
| **CDH6 functional partners** | |
| *CTNNA1* | 20.844 |
| *CTNNB1* | 21.3256197 |
| *CTNND1* | 23.699 |
